# Single cell RNA-seq analysis identifies a noncoding RNA mediating resistance to sorafenib treatment in HCC

**DOI:** 10.1186/s12943-021-01473-w

**Published:** 2022-01-03

**Authors:** Kevin Zhou, Romario Nguyen, Liang Qiao, Jacob George

**Affiliations:** Storr Liver Centre, Westmead Institute for Medical Research, University of Sydney and Westmead Hospital, Westmead, NSW 2145 Australia

## Main text

Sorafenib, a multikinase inhibitor, is an FDA-approved first line drug for the treatment of patients with unresectable hepatocellular carcinoma (HCC) [1]. Unfortunately, these patients achieve minimal therapeutic benefit with an improvement in overall survival of only 3 months [2]. After an initial response, the majority of HCC patients develop drug resistance and disease progression [3]. Although multiple mechanisms such as crosstalk involving PI3K/Akt and MAPK/ERK pathways or activation of EMT have been reported [4, 5], the major drivers of sorafenib resistance remain obscure. Recently, rapidly evolving single-cell transcriptome analysis techniques have been used to comprehensively assess the genetics of the tumour microenvironment and the mechanisms for intra-tumoral heterogeneity which can impact anti-cancer treatment responses [6, 7]. This technology has the potential for higher transcriptomic resolution and sensitivity than bulk sequencing techniques [6, 7]. Single-cell analysis also enables a deep interrogation of hundreds of cancer drivers and identifies individual cells or genes with specific genetic modifications or expression profiles which could contribute to the development of therapeutic resistance. Here, we conducted a single cell RNA seq analysis in sorafenib resistant HCC cells to systematically investigate drug-resistance signatures to uncover the mechanisms of sorafenib resistance.

## Results and discussion

**Fig. 1:**
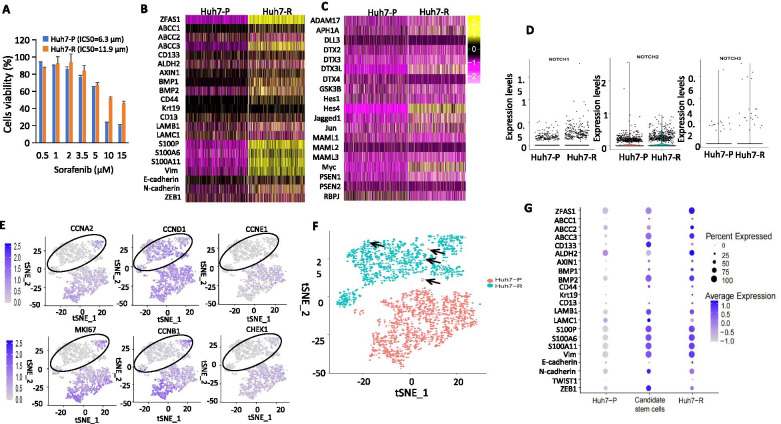
Single-cell enrichment of stemness/EMT gene signatures in sorafenib resistant cells. **A** IC50 of Huh7 parental (Huh7-P) and sorafenib-resistant (Huh7-R) cells. **B-C** Heatmaps showing the expression profiles of stemness/EMT (**B**) and Notch signalling related genes (**C**) as determined by single cell-RNA seq analysis. **D** Scatter plot showing the single-cell expression levels of Notch1–3 receptors in Huh7-P and Huh7-R cells. **E** t-SNE plots along with single-cell expression levels indicated on a purple/grey colour scale of cell-cycle related genes. The black circle represents the Huh7-R cell population with low gene expression. **F** tSNE plot representing the distribution of Huh7-P (red) and Huh7-R (green) cells. Arrows indicate a few Huh7-P cells (candidate stem cells) that are clustered together with Huh7-R cells. **G** Dot-plot showing the expression levels of stemness/EMT genes in Huh7-P candidate stem cells and Huh7-R cells

We successfully established Hep3B and Huh7 sorafenib resistant cells (Hep3B-R and Huh7-R) following long-term exposure of sorafenib to parental Hep3B and Huh7 (Hep3B-P and Huh7-P) cells. The IC50 of Hep3B-R and Huh7-R cells was nearly 2-fold and 2.3-fold higher than that of Hep3B-P and Huh7-P cells (Fig. 1A and Supplementary Fig. 1A). Interestingly, Hep3B-R and Huh7-R cells had greater self-renewal capacity developing significantly larger and more numerous tumour-spheres than the Huh7-P cells (Supplementary Fig. 1B, C). Putative liver cancer stem-like cell populations (LCSCs; defined as EpCAM^+^CD133^+^ cells) were found to be significantly increased in both Hep3B-R and Huh7-R cells (Supplementary Fig. 1D). To assess the enrichment of stem-like phenotypes, nanostring analysis was performed to investigate the targeted mRNA expression of 21 stemness related genes (eg., ALDH1A1, ALDH2, ABCC1, CD133, CD44, EpCAM, Oct4, Sox2, β-catenin). As expected, marked to moderate upregulation of majority of these genes was observed in Hep3B-R and Huh7-R cells (Supplementary Fig. 1E). Consistently, we observed that protein levels of EMT or stemness markers like Vimentin, N-cadherin, Notch2, Notch3, AFP and Oct4 were all upregulated, whereas E-cadherin was downregulated in Hep3B-R and Huh7-R cells (Supplementary Fig. 1F). These data indicate that the HCC cells likely undergo EMT and this is associated with the acquisition of stemness features in response to sorafenib treatment.

The enrichment of stemness/EMT phenotypes at the population level prompted us to hypothesize that their induction might be a major contributor to sorafenib resistance. To test this hypothesis, single-cell RNA sequencing was performed in Huh7-P and Huh7-R cells using the 10X Genomic Chromium System. After stringent filtering of low-quality cells, 4776 cells were selected for subsequent single cell sequencing data analysis. The distinct gene expression signatures between Huh7-P and Huh7-R are visualised in two heatmaps (Fig. 1B, C) and a two-dimensional tSNE plot (Fig. 1F). As shown, Huh7-R cells showed upregulated expression of genes related to stemness and EMT, including multidrug resistant genes (eg., ABCC1, ABBC2, ABBC3), stemness markers (eg., ALDH2, CD13, CD44, CD133, Krt19) and EMT-related genes (eg., LAMB1, LAMC1, S100P, S100A6, S100A11, Vimentin, N-cadherin, ZEB1) (Fig. 1B). It is now understood that Notch signaling is one of the most activated pathways in cancer, playing a central role in cancer progression, EMT promotion and the activation of CSCs [8, 9]. Our data also confirmed that the mRNA expression of Notch receptors (Notch2–4), ligands (DLL1, DLL4, Jagged1) and Notch transcriptional genes (Hes1, Hey1) are all upregulated in patient-derived HCCs (*n* = 12) as compared to matching normal liver, whereas the Notch inhibitor DLL3 was downregulated (Supplementary Fig. 2). Huh7-R cells also exhibited enhanced expression of Notch1–3 receptors (Fig. 1D) and various Notch activators including its ligands (Jagged1), Deltex genes (DTX2–4), transcriptional genes (Hes1, Hes4, MAML, ADAM17), GSK3β and γ-secretase complex components (PSEN1, PSEN2) (Fig. 1C). This was accompanied by downregulation of the key Notch repressors DLL3 and RBPJ (Fig. 1C). This indicated that the activation of Notch signaling serves a crucial role in the induction of stemness/EMT traits and the acquisition of sorafenib resistance.

Despite showing upregulation of stemness/EMT genes, Huh7-R cells showed downregulation of the proliferative marker MKI67, and cell-cycle related genes (Fig. 1E) suggesting that sorafenib treatment may enrich a subset of resistant cells with slow cell cycle or those that lie in a dormant state. This is consistent with the phenomenon of quiescent stem-like cells being maintained in a dormant or slow-dividing state, but subsequently expanding and dividing following cancer treatments [10]. To further confirm the presence of these cells, we identified a small fraction of Huh7-P cells (as indicated by black arrows) which were clustered together with the population of Huh7-R cells (Fig. 1F). We next examined the expression of stemness/EMT genes in these individual cells. Surprisingly, these rare Huh7-P cells (named as candidate stem cells) showed gene expression signatures with great similarity to that of Huh7-R cells (Fig. 1G) suggesting that drug-resistant cells with stemness/EMT traits may have pre-existed in the naïve cells and become prevalent following long-term sorafenib exposure.

Among the genes related to sorafenib resistance, we focused on ZNFX1 antisense RNA 1 (ZFAS1) which had the highest upregulation in Huh7-R cells. ZFAS1 as a novel regulator IncRNA was found to be overexpressed in HCC (*n* = 10) (Supplementary Fig. 3A) and is reported to be involved in tumour growth and metastasis in multiple cancers [11, 12]. We therefore determined the correlation of ZFAS1 expression and clinicopathological features in HCC by analysing publicly available datasets. In this analysis, we found that ZFAS1 expression was significantly upregulated in HCC and recurrent tumours compared with that of matching normal liver by using the GEPIA dataset (Supplementary Fig. 3B) and the University of California Santa Cruz (UCSC) Xena project (http://xena.ucsc.edu) (Supplementary Fig. 3C). Moreover, ZFAS1 was overexpressed at higher stages (III/IV) of HCC and in poorly (G3)/undifferentiated HCC (G4) compared with those at early stages (I/II) (Supplementary Fig. 3D) and in highly (G1)/moderately (G2) differentiated HCC (Supplementary Fig. 3E). By analysing the GEPIA dataset, higher ZFAS1 expression levels were associated with shorter overall survival (OS, Supplementary Fig. 3F) and disease-free survival (DFS, Supplementary Fig. 3G) suggesting that ZFAS1 may serve as a novel prognostic marker of HCC. To clarify the role of ZFAS1 in the induction of stemness and EMT phenotypes, we used the cBioportal dataset to investigate the correlation of ZFAS1 and stem-ness/EMT genes. As expected, ZFAS1 expression was positively correlated with the expression of multiple stemness genes (eg., EpCAM, CD24, CD90, CD133, DLK1, Krt18/19) and EMT markers (eg., S100A4/A6, Twist, Vimentin) (Supplementary Fig. 4).

**Fig. 2:**
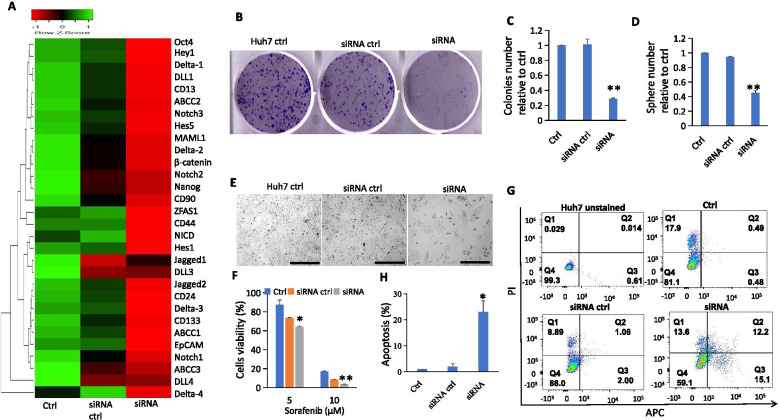
Role of ZFAS1 in the induction of stemness and sorafenib resistance. **A** Bulk mRNA expression levels of stemness and notch signaling related genes in Huh7 cells with or without ZFAS1 transfection presented as a heatmap. **B** Representative images of tumour colony of Huh7 cells with or without siRNA transfection. **C-D** Quantitative analysis of tumour colonies (**C**) and sphere numbers (**D**). Data represented as mean ± standard deviation (SEM). **: *p* < 0.01 (compared to Ctrl). **E** Representative images of cell growth of Huh7 cells with or without siRNA transfection. Scale bar = 100 μm. **F** Viability of Huh7 cells with or without siRNA transfection following sorafenib (5 μM and 10 μM) treatment. Data represented as mean ± SEM. *: *p* < 0.05; **: *p* < 0.01 (compared to Ctrl). **G** Representative images of apoptotic percentage of Huh7 cells with or without siRNA knockdown after sorafenib treatment. **H** Quantitative analysis of **G**. Data represented as mean ± SEM. *: *p* < 0.05 (compared to Ctrl)

To investigate whether blockade of ZFAS1 could impair HCC progression and stemness properties, Huh7 cells were transfected with ZFAS1-specific siRNA and ~ 80% ZFAS1 knockdown was achieved (Fig. 2A). Knockdown of ZFAS1 significantly downregulated mRNA expression of various stemness (eg., ABCC1–3, CD13, CD24, CD44, CD90, CD133, EpCAM, Nanog, Oct4) and notch signaling pathway related genes (eg., Notch1–3, Delta1–4, Jagged2, Hes1/Hey1, DLL1, DLL4) (Fig. 2A). ZFAS1 siRNA-transfected Huh7 cells also formed significantly fewer and smaller tumour colonies (Fig. 2 B,C) and spheres (Fig. 2D) compared to untreated cells or cells transfected with control (ctrl) siRNA. This suggests that ZFAS1 might play a crucial role in HCC progression and self-renewal. Previous studies have reported that silencing ZFAS1 enhances the chemo-sensitivity of gastric, ovarian and cervical cancers [13–15]. To validate whether silencing ZFAS1 could sensitize sorafenib treatment, Huh7 cells with or without ZFAS1 siRNA transfection were treated with sorafenib. As expect, sorafenib treatment significantly inhibited cells growth (Fig. 2E) and reduced cell viability (Fig. 2F) in ZFAS1-silenced Huh7 cells compared to untreated ctrl cells. Knockdown of ZFAS1 also caused significantly higher apoptosis in Huh7 (Fig. 2G and H). All these data indicate that ZFAS1 is a key mediator of sorafenib resistance.

## Conclusions

In summary, this study has demonstrated the precise role of stemness and EMT in driving sorafenib resistance at the single cell level. A small fraction of quiescent stem-like cells with intrinsic sorafenib resistance pre-exists in the HCC cell population and becomes enriched following long-term sorafenib exposure. This data provides new insights indicating that dormant stem-like cells are possibly the main culprits facilitating the development of sorafenib resistance. Therefore, killing quiescent cancer stem-like cells might be a strategy to overcome sorafenib resistance. We also identified a novel noncoding lncRNA, ZFAS1 which is closely associated with HCC proliferation and the maintenance of stemness characteristics. Silencing ZFAS1 is thus another strategy to overcome sorafenib resistance.

## Supplementary Information

**Additional file 1.**

**Additional file 2.**

## Data Availability

All data and materials generated in this study are available from the Corresponding author upon reasonable request.

## Abbreviations

ABCC: ATP binding cassette subfamily C
ALDH: Aldehyde dehydrogenase
AXIN1: Axis inhibition protein 1
BMP: Bone morphogenetic protein
DLL: Delta-like protein
EMT: Epithelial-to-mesenchymal transition
ERK: Extracellular regulated protein kinase
HCC: Hepatocellular carcinoma
Hes: Hairy and enhancer of split homolog
IncRNA: Long non-coding RNA
LAMA1/B1/C1: Laminin subunit alpha/Beta/Gamma 1
MAML: Mastermind-like
MAPK: Mitogen-activated protein kinase
PI3K: Phosphoinositide 3-kinase
Vim: Vimentin
ZEB1: Zinc finger E-Box homeobox 1
ZFAS1: zinc finger antisense 1
