# Supplementary material for: Single cell RNA-seq analysis identifies a noncoding RNA mediating resistance to sorafenib treatment in HCC
Source: Mol Cancer. 2022 Jan 3;21:6. doi: 10.1186/s12943-021-01473-w (PMC8722008; doi:10.1186/s12943-021-01473-w)
Supplement: Supplementary file 1 — Additional file 1. [file 12943_2021_1473_MOESM1_ESM.docx]

**Single cell RNA-seq analysis identifies a noncoding RNA mediating resistance to sorafenib treatment in HCC**

Kevin Zhou^1^, Romario Nguyen^1^, Liang Qiao^1^, Jacob George^1^

^1^Storr Liver Centre, Westmead Institute for Medical Research, University of Sydney and Westmead Hospital, Westmead, NSW 2145, Australia

**Methods and Materials**

**Cells and reagents**

Liver cancer cell lines Hep3B and Huh7 were purchased from American Type Cell Collection (ATCC, VA, USA). Hep3B and Huh7 cells were grown in Dulbecco’s Modified Eagle Medium (DMEM) (Lonza) supplemented with 10% fetal bovine serum (FBS) at 37°C in 5% CO_2_. Sorafenib were obtained from AbMoles Bioscience (Burlington, NC, USA) and dissolved in dimethyl sulfoxide (DMSO) to make a stock concentration of 100 mM.

**The generation of sorafenib resistant Huh7 cells**

Hep3B and Huh7 sorafenib resistant cells (Hep3B-R and Huh7-R) were generated through long-term exposure of cells to incremental concentrations of sorafenib. After treating with various concentrations of sorafenib, cells viability was determined by cell counting kit 8 (CCK8) according to manufacturer’s instruction. The half maximal inhibitory concentration (IC50) was calculated using the equation of “Dose-Response-Inhibition” of Graphpad Prism 8.

**Quantitative Reverse Transcript-Polymerase Chain Reaction and Western-blot**

Total RNA and protein from parental and sorafenib resistant cells were extracted. cDNA was then amplified using CFX384TM Real-Time System (Bio-Rad, California, USA). The mRNA expression levels of targeted genes were normalised using the ΔΔCt methods as described previously [1]. Western-blots were performed as we previously reported [2].

**Library construction and sequencing**

Huh7 cell suspensions were harvested and stained with Totalseq-A Hashtag antibody-oligo conjugates (Biolegend) according to the manufacturer’s instruction. The labelled samples were combined in pairs in equal ratio (5,000 cells per sample) and then loaded into a Chromium B Chip (10X Genomics) for target output of 10,000 cells per reaction. The 3’ cDNA library and Hashtag Oligo (HTO) library was constructed by Chromium Controller (10X Genomics) based on the Chromium Single-Cell 3’Reagent Kit v3 protocol (Rev D) with minor modifications. Briefly, HTO additive primers were mixed with cDNA amplification reagents to simultaneously amplify cDNA and TotalSeq HTO tags. SPRI select beads (Beckman Coulter) were used to separate the amplified cDNA and HTO tags followed by the post-amplification clean-up. Size and concentration of amplified libraries were assessed by Agilent Tapestation 4200 System and then pooled (95% cDNA library, 5% HTO library). Library pools were then sequenced by a Novaseq 6000 system (Illumina) using 100bp paired-end sequencing at 28,500 reads/cell for cDNA libraries and 1,500 reads/cell for HTO libraries.

**Single cells sequencing data analysis**

Cell barcode/RNA counts were generated by using Cellranger (v3.0.2) and 4776 cells were estimated from the barcode distribution. By using Cellranger barcodes as a whitelist and a maximum hamming distance 1, Hash libraries were then counted using CITESeq-count (<https://github.com/Hoohm/CITE-seq-Count>). In brief, cells in individual runs were identified using the HTODemux function of cell hashtags. Only barcodes which were identified as singletons and had less than 25% mitochondrial RNA reads were selected for further analysis (1645 parental and 1713 resistant cells). Data for each treatment was normalized and scaled before clustering based on nearest neighbour analysis of the first 20 dimensions for the top 2000 variably expressed genes. tSNE projections using default parameters were used for visualisation. Gene expression values were plotted using Seurat Plot functions.

**The transfection of siRNA, tumor sphere, colony formation and Annexin V APC-PI apoptosis assay**

Huh7 cells were cultured in 6-well plate (1×10^5^ cells/well) and then transfected with 30 pM of anti-ZFAS1 siRNA (Catalogue No: AM16708, ThermoFisher) using the Lipofectamine 2000 transfection reagent (Invitrogen) according to the manufacturer’s instructions. SiRNA and transfection reagents were all diluted in Opti-MEM (Gibco). Huh7 cells with or without ZFAS1 siRNA transfection were subjected to tumour sphere and colony formation assay as we previously reported [1,2].

Huh7 cells with or without siRNA transfection were cultured in 96-well (3000 cells/well) or 6-well plates. Cells in 96-well plate were treated with 5 µM or 10 µM sorafenib for 72 h and cells viability were determined by CCK8 kit. Cells in 6-well plate were treated with 5 µM sorafenib and harvested for Annexin-V/PI double staining analysis as we previously described [2].

**Supplementary figure legends**

**Supplementary Fig.1** Bulk enrichment of stemness/EMT traits of Hep3B-R and Huh7-R cells. **A** IC50 of Hep3B-P and Hep3B-R cells. **B** Representative images of tumour spheres from Hep3B and Huh7 parental and resistant cells. **C** Quantitative analysis of B. **D** EpCAM+CD133+ cell populations determined by flow cytometry. Data represented as mean ± standard deviation (SEM). *: *p*<0.05; **: *p*<0.01 (compared to parental cells). **E** Targeted stemness related genes mRNA expression as determined by Nanostring; **E** Protein expression of EMT and stemness related genes as determined by Western-blot.

**Supplementary Fig.2** The bulk mRNA expression of Notch receptors (Notch1,2,3,4), ligands (DLL1, DLL3, DLL4, Jagged1) and transcriptional genes (Hes1, Hey1) in HCC tumours and adjacent normal liver (n=12) as determined by qPCR.

**Supplementary Fig.3** Correlation of ZFAS1 with HCC development, metastasis and prognosis. **A** ZFAS1 expression in HCCs and matching adjacent normal liver (n=10) tested by qPCR. **B** Data from the GEPIA dataset showing ZFAS1 expression in HCC and normal liver. **C** Data from the UCSC Xena project (<http://xena.ucsc.edu>) showing ZFAS1 expression in normal liver, adjacent liver, primary and recurrent HCC tissues. **D-E** ZFAS1 expression in HCCs at different differentiation (UCSC Xena project) and clinical stages (GEPIA dataset). **F-G** Overall and disease-free survival of HCC patients with low and high ZFAS1 expression (GEPIA dataset).

**Supplementary Fig.4** Spearman correlation between ZFAS1 expression and the expression of stemness and EMT related genes. Raw data were obtained from the cBioportal database.

**References**:

1. Wilson GS, Hu ZN, Duan W, Tian AP, Wang XM, Mcleod DC, Lam V, George J, Qiao L. Efficacy of using cancer stem cell markers in isolating and characterizing liver cancer stem cells. Stem Cells Dev. 2013;22(19):2655-64.
2. Wilson GS, Tian AP, Hebbard L, Duan W, George J, Li X, Qiao L. Tumoricidal effects of the JAK inhibitor Ruxolitinib (INC424) on hepatocellular carcinoma *in vitro*. Cancer Lett. 2013;341(2):224-30.
