# Supplementary figures and images for: Single cell RNA-seq analysis identifies a noncoding RNA mediating resistance to sorafenib treatment in HCC
Source: Mol Cancer. 2022 Jan 3;21:6. doi: 10.1186/s12943-021-01473-w (PMC8722008; doi:10.1186/s12943-021-01473-w)

# Supplementary Figure 1

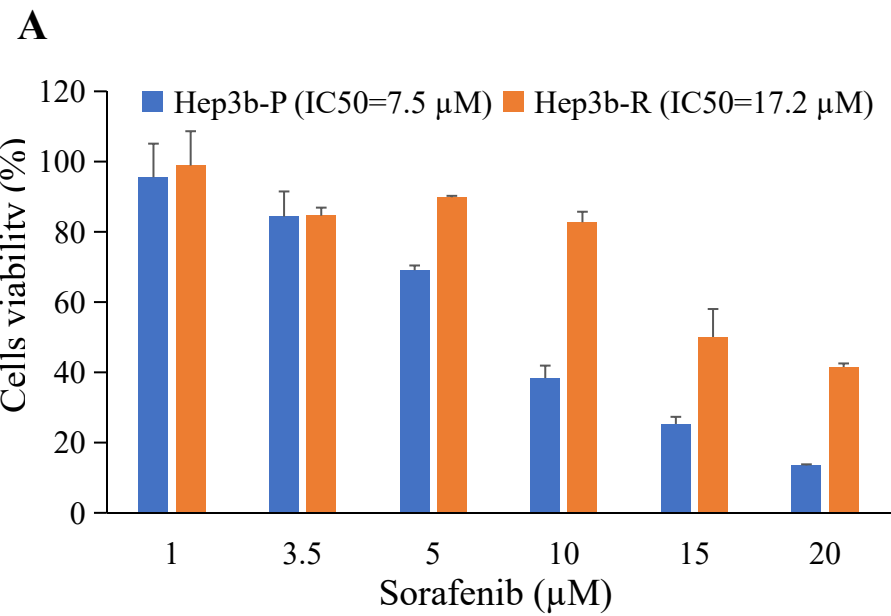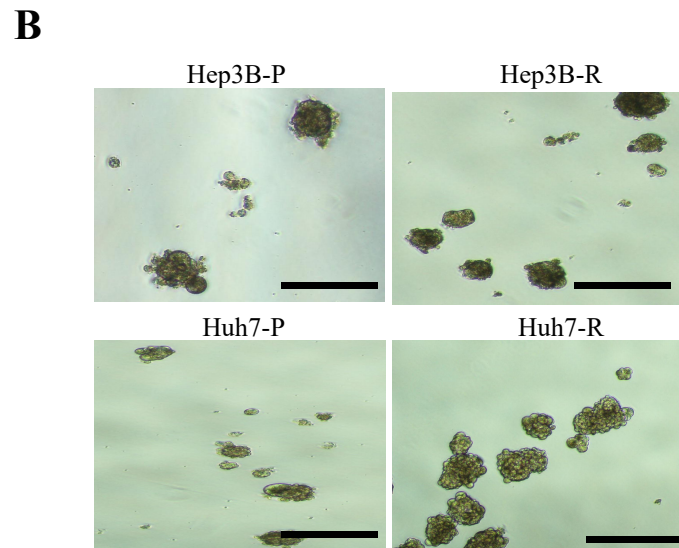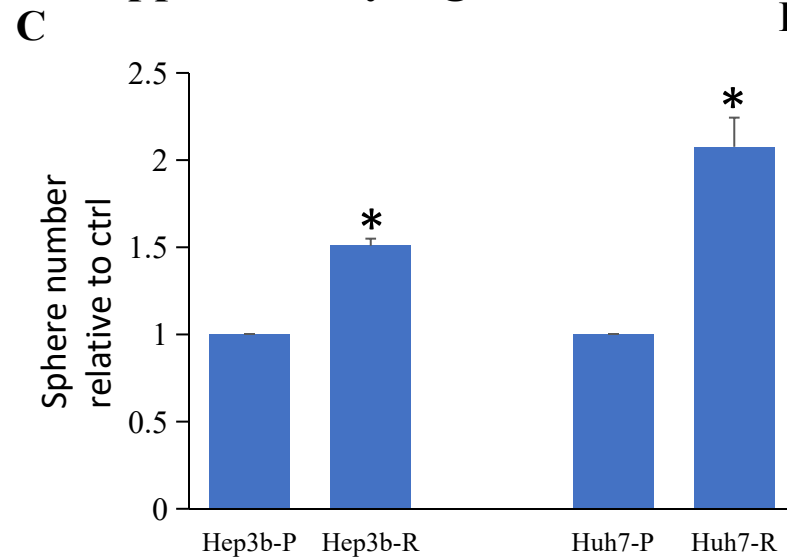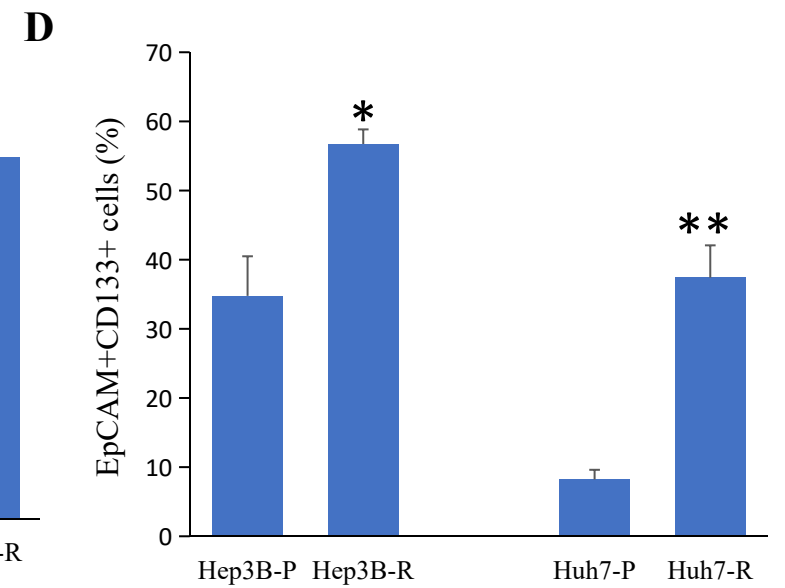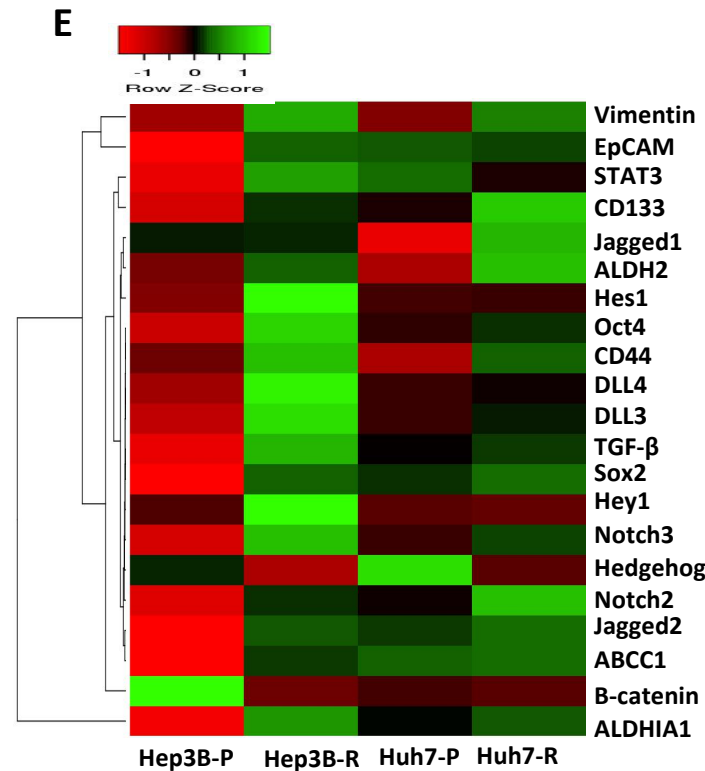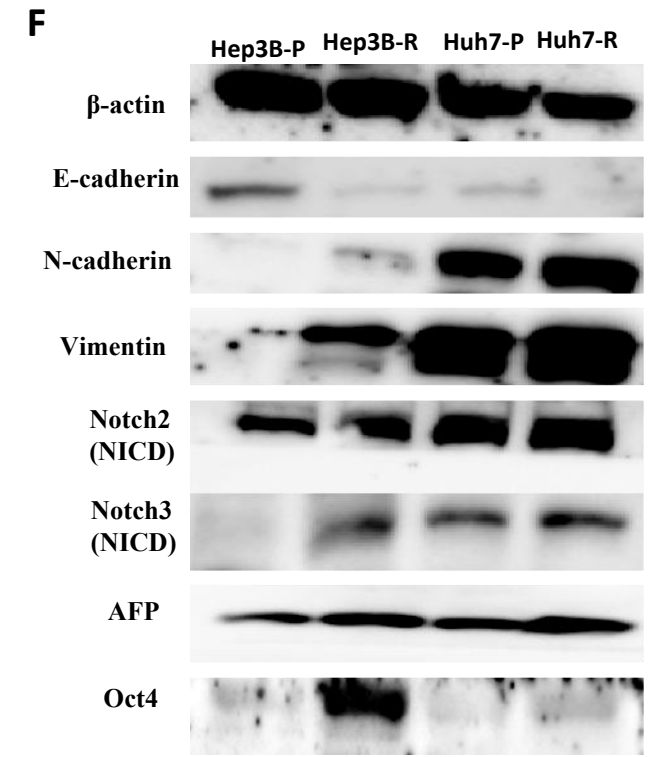

# Supplementary Figure 2

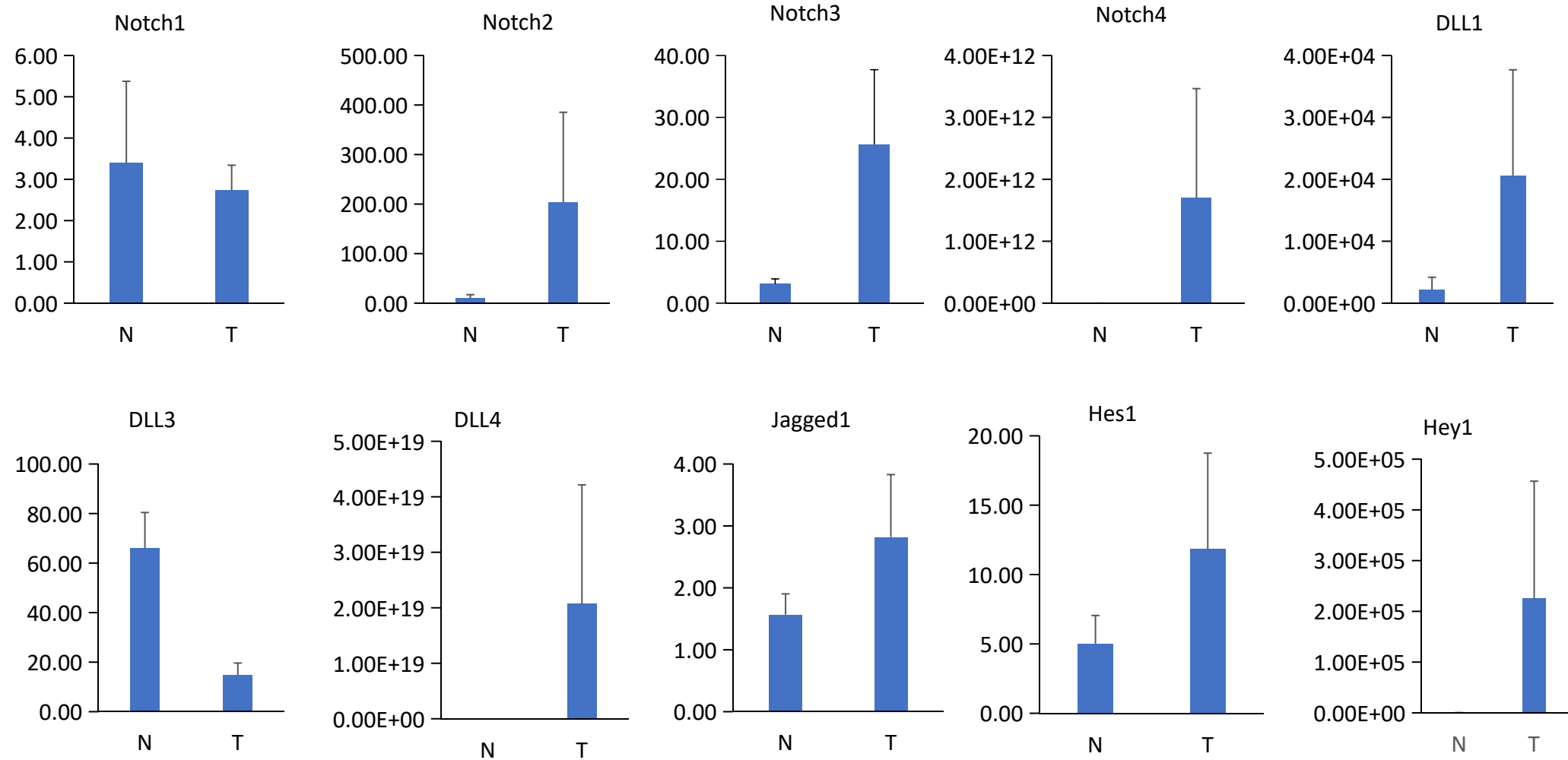

Supplementary Figure 3

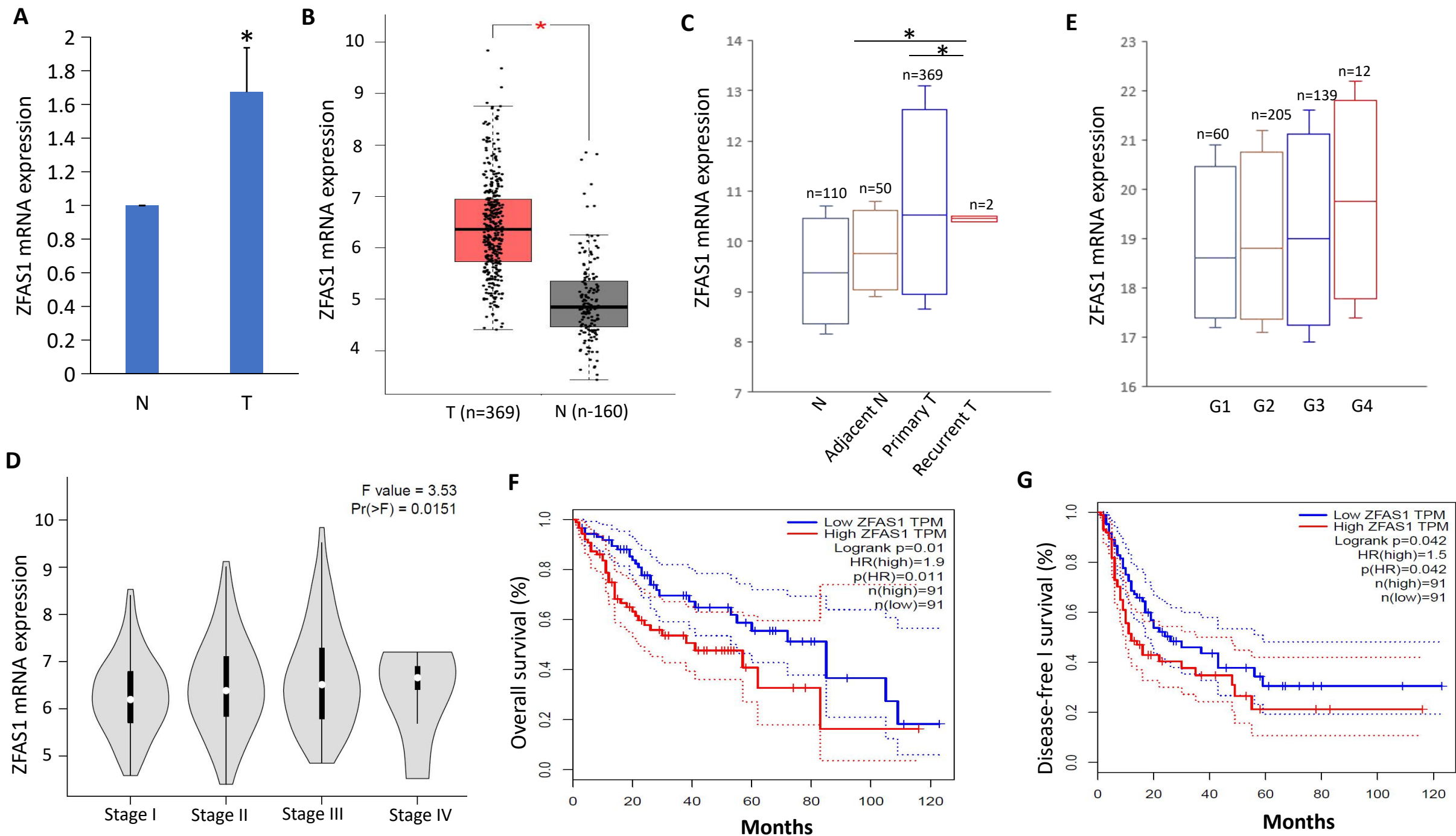

## Supplementary Figure 4

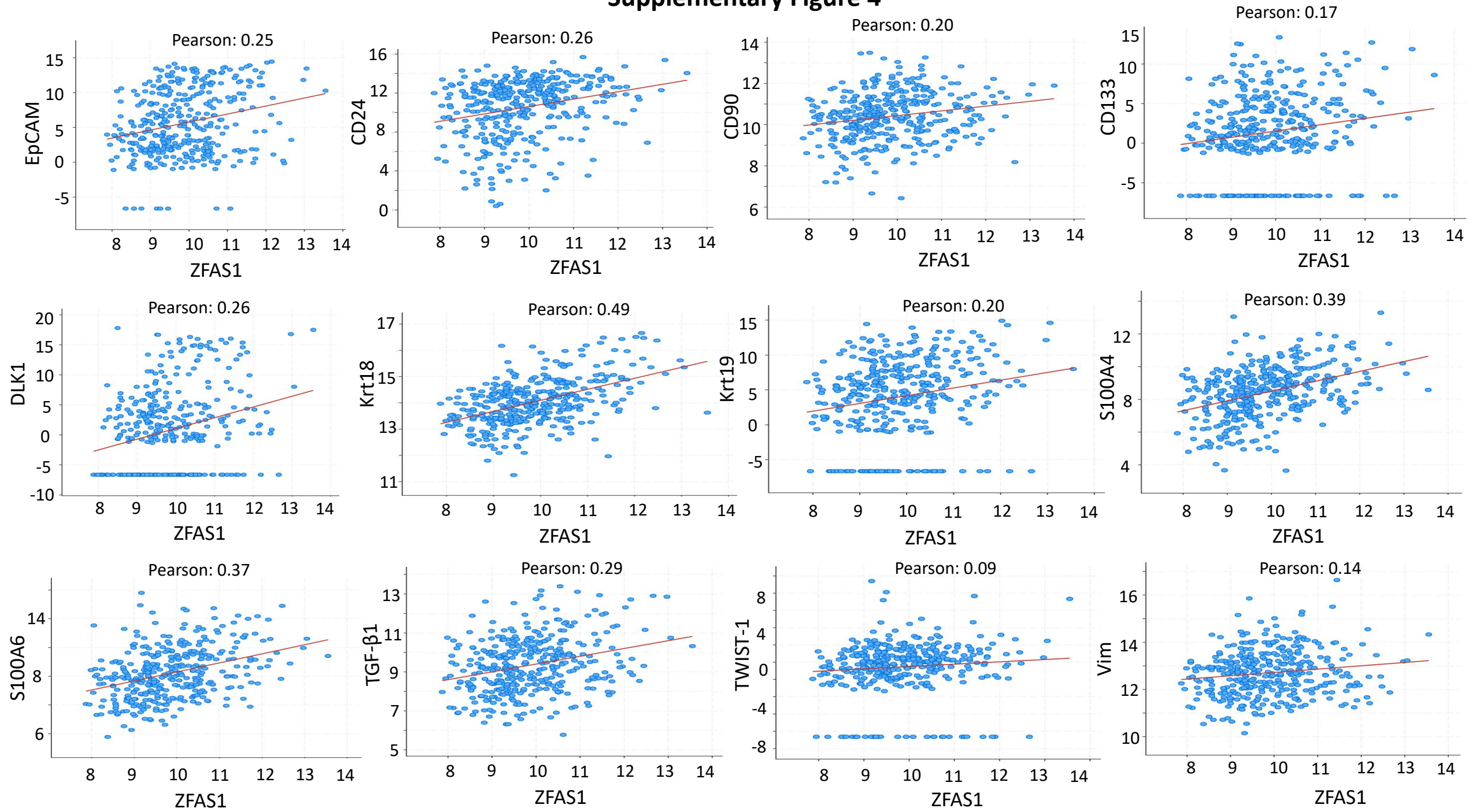

Supplement: Supplementary file 2 — Additional file 2. [file 12943_2021_1473_MOESM2_ESM.pdf]
